# Improved early recovery and shorter hospital stay with fast track protocol versus standard care in total hip arthroplasty: 5‐year results from a prospective randomised controlled study

**DOI:** 10.1002/jeo2.70528

**Published:** 2025-12-28

**Authors:** Pietro Cimatti, Martina Rocchi, Benedetta Dallari, Nicolandrea Del Piccolo, Alessandro Mazzotta, Dante Dallari

**Affiliations:** ^1^ Reconstructive Orthopaedic Surgery Innovative Techniques–Musculoskeletal Tissue Bank IRCCS Istituto Ortopedico Rizzoli Bologna Italy

**Keywords:** anterior hip approach, early rehabilitation, enhanced recovery, fast track surgery, total hip arthroplasty

## Abstract

**Purpose:**

Total hip arthroplasty (THA) is a widely performed surgery with growing demand globally. This study aims to evaluate the effectiveness of a fast track (FT) protocol compared to standard care (SC) in patients undergoing THA.

**Methods:**

Ninety patients aged 18–70 years with primary unilateral hip osteoarthritis, American Society of Anesthesiologists (ASA) score <3, body mass index (BMI) ≤ 32 and no cognitive or psychiatric disorders were prospectively enrolled from March 2018 to January 2020. All patients provided informed consent and were randomised to the FT or SC groups. The FT protocol consisted of preoperative education, oral analgesic pain management and early intensive rehabilitation. Functional autonomy was assessed on postoperative Day 3 using the Iowa Level of Assistance (ILOA) scale. Follow‐up assessments at 6 weeks, 3, 6, 12 and 60 months included the Harris Hip Score (HHS) and the Western Ontario and McMaster Universities Osteoarthritis Index (WOMAC).

**Results:**

Forty‐six patients were assigned to the FT group. Both groups were comparable in baseline demographics. The FT group showed significantly faster early functional recovery, with lower ILOA scores on postoperative Day 3 (9.60 ± 5.2 vs. 11.7 ± 3.4; *p* = 0.024), and a shorter hospital stay (3.54 ± 1.25 vs. 6.39 ± 1.59 days; *p* < 0.0001). WOMAC scores were significantly better in the FT group at 6 weeks (10.38 ± 9.18 vs. 14.21 ± 8.76; *p* = 0.035) and remained superior at 60 months (0 ± 0 vs. 0.27 ± 0.81; *p* = 0.027). Although baseline HHS was higher in the FT group, greater improvements from baseline were seen in the SC group at later follow‐ups, likely due to a ceiling effect.

**Conclusions:**

The FT protocol enhances early postoperative recovery and significantly reduces hospital stay after THA without compromising safety. Long‐term functional outcomes favour the FT approach, supporting its implementation to improve recovery in appropriately selected patients undergoing hip arthroplasty.

**Level of Evidence:**

Level II.

**Abbreviations**
ASA: American Society of Anesthesiologists
BMI: body mass index
FT: fast track
HHS: Harris hip score
ILOA: Iowa level of assistance
SC: standard care
THA: total hip arthroplasty
TXA: tranexamic acid
WOMAC: Western Ontario and McMaster Universities Osteoarthritis Index

## INTRODUCTION

Total hip arthroplasty (THA) has become a highly successful surgical procedure, with thousands of surgeries performed annually worldwide [27]. It is particularly effective in treating hip osteoarthritis and provides excellent long‐term outcomes [26]. The rising demand for hip arthroplasty can be attributed to several factors, including population ageing, increased life expectancy and arthritic conditions resulting from injury or physical trauma [1, 11].

Over the past decades, significant advancements have been made in biomechanics, tribology, prosthesis design and surgical techniques. However, these innovations have not been matched by an equivalent progress in perioperative care pathway.

In this context, fast postoperative recovery has emerged as a critical component of patient management. It is essential to balance cost containment in healthcare with maintaining high standards of patient care, satisfaction and improved patient‐reported outcomes [3]. Consequently, the focus of research has shifted toward the quality of outcomes and the patient′s subjective experience. Accelerating recovery during the postoperative period has become a key objective, representing the integration of the latest surgical and medical best practices. ‘Fast‐track’ (FT) surgery was introduced as a coordinated multidisciplinary approach aimed to reducing surgical stress and enhancing postoperative recovery. This strategy emphasises not only early discharge but also the overall improvement in patient recovery. Although FT surgery was developed in the 1990s, its adoption in the orthopedical practice was initially slow, due to challenges such as variability in hospital resources and insufficient postoperative mobilisation protocols. The focus of the scientific community has shifted towards not only clinical outcomes but also their quality and subjective patient experience. Rapid recovery during the postoperative phase has become a crucial goal, representing a synthesis of the most advanced surgical and medical practices aimed to minimising surgical stress and enhancing recovery. FT surgery, also known as enhanced recovery after surgery, was introduced in the 1990s as a coordinated multidisciplinary approach designed to reduce surgical stress, improve pain management and facilitate early mobilisation to accelerate postoperative period. This approach leads not only to early discharge but also to an overall improvement in patient recovery and satisfaction. Although FT surgery was developed decades ago, its implementation in orthopaedic clinical practice has been relatively slow, largely due to challenges such as variability in hospital infrastructure and protocols, as well as issues with adequate postoperative patient mobilisation and adherence to rehabilitation protocols [21, 23, 24].

The main goal of the present randomised clinical trial was to evaluate whether our FT THA protocol resulted in better outcomes than a regular protocol in patients who underwent primary THA. The primary hypothesis is that the adoption of a FT protocol in patients undergoing THA results in significantly faster postoperative functional recovery compared to standard clinical care (SC). The secondary hypothesis is that the FT protocol significantly reduces the length of hospital stay (LOS) in comparison to the SC protocol.

## MATERIALS AND METHODS

### Study design

This single‐centre, randomised controlled clinical trial was conducted in a specialised orthopaedic unit dedicated to hip prosthetic surgery. Patients referred for this trial are screened for eligibility according to the principle of inclusion and exclusion criteria, as reported in Table 1. A total of 90 patients meeting the eligibility criteria were prospectively enrolled between March 2018 and January 2020. One patient withdrew from the study due to personal reasons. All participants provided written informed consent before enrolment and were randomly assigned to either the FT group or the SC group using a computer‐generated randomisation list. Group allocation was concealed until the intervention was assigned.

**Table 1:** Inclusion and exclusion criteria.

Inclusion criteria	Exclusion criteria
Age between 18 and 70 years, both sexes	Lack of written consent of study participation
ASA score ≤ 2	Patients with cognitive impairment and psychiatric disorders
BMI < 32	Rheumatoid arthritis
Primary hip osteoarthritis or secondary hip osteoarthritis due to femoroacetabular impingement	Previous hip surgery
Preoperative hemoglobin levels >13 g/dL	Presence of contraindications to spinal anaesthesia
Presence of a caregiver	Absence of a caregiver

Abbreviations: ASA, American Society of Anesthesiologists; BMI, body mass index.

To minimise bias, all postoperative functional assessments were conducted by trained physiotherapists who were blinded to the group allocation. Data analysts were also blinded during the statistical analysis phase. All patients were screened for eligibility during routine pre‐admission evaluations. Baseline assessments, including medical history and preoperative functional status (Western Ontario and McMaster Universities Osteoarthritis Index [WOMAC] and Harris hip score [HHS]), were recorded at the pre‐admission visit [8, 30, 34]. Patients who underwent reoperations due to postoperative complications were excluded from subsequent functional evaluations, as their functional outcomes thereafter were attributable to the secondary intervention rather than the primary procedure.

**Figure 1:**
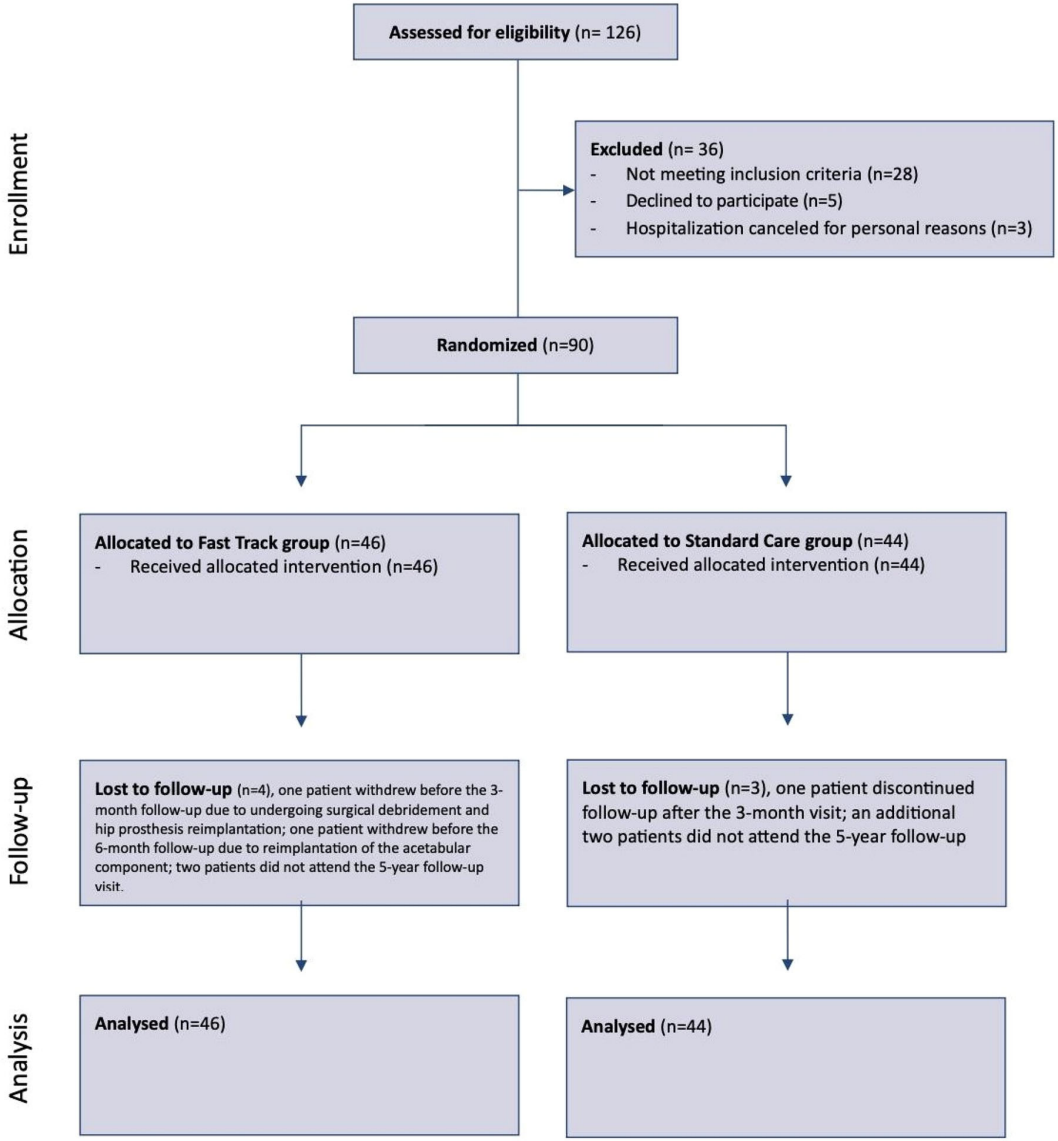
CONSORT flow diagram.

A detailed overview of patient flow throughout the trial, including enrolment, allocation, follow‐up and analysis, is presented in the CONSORT diagram (Figure 1).

### Surgical and perioperative protocols

All patients underwent minimally invasive THA using standardised components: short, uncemented stems (Pulchra–Adler Ortho), hemispherical acetabular cups (Fixa Ti‐Por–Adler Ortho) and ceramic‐on‐ceramic articulations. To minimise variability, all procedures were performed by the same experienced orthopaedic surgeon. In our study, minimally invasive surgery was performed using a muscle‐sparing anterior approach, which has been well‐documented in the literature for its advantages in reducing surgical trauma, blood loss and postoperative pain, while promoting faster functional recovery [1, 2, 3]. This approach utilises a smaller skin incision (usually 8–12 cm) and avoids detachment or cutting of major muscle groups around the hip, particularly preserving the gluteal muscles and minimising disruption of the joint capsule [4]. This technique is associated with reduced intraoperative bleeding, lower postoperative pain scores and shorter hospital stays compared to traditional posterior or lateral approaches [5, 10]. Control of erythropoiesis in our perioperative management refers to the strategies implemented to monitor and optimise patients′ red blood cell production and minimise blood loss, thereby reducing the need for allogeneic blood transfusions. This includes preoperative assessment of hemoglobin levels and any iron reserves corrected preoperatively through iron administration, and intraoperative use of tranexamic acid to reduce bleeding, and postoperative monitoring of haematologic parameters to detect anaemia early [6, 7, 9, 36]. Maintaining effective erythropoiesis and minimising blood loss is crucial in THA to improve recovery outcomes and reduce complications related to transfusions [35].

In particular, the tranexamic acid administration schema adopted for all patients except complications is based on 1 g at induction, 1 g 1 h after the beginning of the surgery and 1 g after 6 h.

We have now included these clarifications in the revised manuscript to ensure precise understanding of our surgical and perioperative protocols.

### Rehabilitation protocol

In detail, the rehabilitation protocols adopted in FT and SC groups are shown in Table 2. This protocol follows a gradual progression in postoperative rehabilitation, to have the patient resume function and independency as soon as possible after surgery.

**Table 2:** Fast track versus standard care rehabilitation protocol.

Rehabilitation	Fast track	Standard care
Surgical operation	Day 0	Surgical operation
Mobilisation in bed	Day 0	Day 1
Resumption of verticalization ambulation with the assistance of physical therapists and an anteriorwalker	Day 0	Day 1
Stairs climbing Gradual weaning from the use ofcrutches	Day 1	Day 3–4
Gradual resumption of ambulation Discharge of the patient	Day 3	Day 5–7

### Analgesia protocols

Both FT and SC groups received evidence‐based perioperative pain management protocols consistent with current best practices in THA. Patients in the FT group received a multimodal analgesic regimen that included preoperative gabapentin, nonsteroidal anti‐inflammatory drugs (NSAIDs) and oral rescue opioids (e.g., oxycodone or tapentadol) if the Numeric Rating Scale (NRS) was ≥3. This protocol aimed to minimise opioid use and promote early mobilisation, as supported by recent literature [1, 2].

In the SC group, pain management followed the institution′s conventional regimen, consisting of intravenous ketorolac and tramadol every 8 h. Rescue analgesia included oxycodone as needed and IV paracetamol (1000 mg every 8 h). This standard approach has demonstrated effectiveness in controlling postoperative pain while limiting excessive opioid use [3, 4] (Table 3).

**Table 3:** Perioperative pain management in fast track and standard care protocol.

Pain management	FT	SC
2 h before surgery	1 tablet of paracetamol 1000 mg	
	1 tablet of gabapentin 600 mg	
	1 tablet of celecoxib 200 mg	
4 h after surgery	1 tablet of paracetamol 1000 mg	Ketorolac and tramadol every 8 h
	1 tablet of gabapentin 300 mg	
Before sleeping	1 tablet of 10 mg oxycodone or 1 tablet of 100 mg tapentadol	
Day 1	1 tablet of paracetamol 1000 every 6 h	Ketorolac and tramadol every 8 h
	1 tablet of celecoxib 30 mg at 8 AM 1 tablet gabapentin 300 mg at 9 AM	
From Day 2 to a maximum of 15 days	1 tablet of paracetamol 1000 every 12 h and 1 tablet of celecoxib 200 at 8 AM	1 tablet of paracetamol 1000 every 12 h and 1 tablet of celecoxib 200 at 8 AM
Rescue dose	1 tablet of paracetamol/c odeine 500/30 mg (repeatable after 12 h)	Oxycodone as needed and IV paracetamol (1000 mg every 8 h)

Abbreviations: FT, fast track; SC, standard care.

### Statistical analysis

Participants were randomised to either the SC or FT group using the permuted block method implemented through ER software, ensuring balanced allocation between groups. Randomisation was performed centrally at the IRCCS Istituto Ortopedico Rizzoli by an independent colleague who was not involved in patient enrolment and remained blinded to participants, investigators, healthcare providers and outcome assessors. The randomisation sequence was concealed in sequentially numbered, sealed envelopes, which were opened by the enrolling investigator on the day of patient inclusion.

A modified intention‐to‐treat approach was applied for data analysis. Patients were assessed on postoperative Day 3, at discharge and during follow‐up visits at 6 weeks, 3, 6, 12 and 60 months, in accordance with standard clinical practice. The primary outcomes included clinical‐functional scores measured by the WOMAC and HHS, LOS, the number of postoperative blood transfusions and adverse events. The secondary outcome was the early postoperative functional recovery as measured by the Iowa Level of Assistance (ILOA) scale on the third postoperative day. Both evaluators and data analysts were blinded to group assignments. The autonomy level at hospital discharge measured by the ILOA scale, was assessed by experienced physiotherapists at our institution who follow standardised protocols for functional evaluation of patients undergoing fast‐track hip arthroplasty. To minimise measurement bias related to the physiotherapists′ assessment of the ILOA scale, we implemented several measures:

Training and standardisation: All physiotherapists involved received specific training on the ILOA scale, including detailed sessions to standardise the administration and scoring of each functional task (supine‐to‐sitting, sitting‐to‐standing, walking, stair climbing and gait speed). This ensured consistency in the evaluation process.

Assessments performed in routine clinical settings: Evaluations were carried out during regular physiotherapy sessions, thereby reducing the likelihood that assessors were aware of the study′s primary endpoint and thus limiting observer expectation bias.

Partial blinding of evaluators: While complete blinding was not feasible, the physiotherapists performing the assessments were not involved in clinical decisions regarding patient discharge, minimising potential influence of the evaluation on treatment decisions.

Objective discharge criteria: Patients were discharged only upon meeting predefined functional criteria, specifically the ability to safely ascend at least three steps in a communal area. This criterion is a well‐established, clinically relevant threshold of functional independence and serves to limit subjective variability in discharge timing.

The combination of targeted assessor training, standardised evaluation procedures, assessments conducted in a routine clinical context, and clear, objective discharge criteria significantly attenuated the risk of measurement bias in ILOA scoring.

Statistical analyses were conducted using GraphPad Prism version 6. For variables reported with mean and standard deviation, 95% confidence intervals (CI) were also calculated to indicate the range within which the true group mean is expected to fall with 95% CI. Continuous variables are presented as mean ± standard deviation, while categorical variables are expressed as frequency and percentage.

Group comparisons of continuous variables that were normally distributed and homoscedastic were performed using one‐way ANOVA. In cases where these assumptions were not met, the nonparametric Mann–Whitney *U*‐test was applied. Differences in ILOA between groups were also assessed using the chi‐square test. A *p*‐value of less than 0.05 was considered statistically significant.

For primary outcomes (WOMAC, HHS, LOS, blood transfusions and adverse events), the Mann–Whitney *U*‐test was used to compare groups at each time point. To analyse changes over time, the percentage change (Delta) from baseline was calculated and compared between groups. Additional statistical analyses were performed using IBM SPSS Statistics for Windows, Version 19.0 (IBM Corp.), under the guidance of an experienced statistical consultant from our institute.

As a secondary outcome, the change in the ILOA scale score on the third postoperative day was evaluated. Assuming a standard deviation of ±6.9 for the ILOA score, a clinically meaningful difference of seven points, 90% power and a significance level of 0.05, it was estimated that a minimum of 40 patients per group (1:1 allocation) would be required to detect this difference.

## RESULTS

Descriptive analysis of the study sample: a total of 90 patients were enrolled and randomised into two groups: 46 in the FT group and 44 in the SC group. The FT group included 20 females and 26 males, while in the SC group, there were 15 females and 29 males. The distribution by biological sex was not significantly different between groups, as confirmed by Fisher′s exact test (*p* = 0.361). The mean age of the overall cohort was 55 ± 9.5 years. No significant age difference was found between the two groups (56.2 ± 8.5 vs. 54.2 ± 10.5 years; *p* = 0.170, ANOVA). The overall mean body mass index (BMI) was 27.9 ± 2.9 kg/m². A significant intergroup difference was found, with the SC group exhibiting a higher mean BMI (30.2 ± 1.3) than the FT group (25.8 ± 3.4), as confirmed by analysis of variance (ANOVA, *p* < 0.001).

Health status at admission was assessed using the American Society of Anesthesiologists (ASA) classification. No significant difference in ASA score distribution was observed between groups (*p* 233 = 0.317, Pearson′s chi‐square test). At baseline, 26% of patients in the FT group and 35% in the SC group were active smokers (*p* = 0.328).

Before surgery, approximately 11% of patients in both groups had received intra‐articular injections. Primary hip osteoarthritis was the most prevalent diagnosis, occurring in 39 of 46 cases (84.8%) in the FT group and 34 of 44 cases (77.3%) in the SC group. Secondary osteoarthritis was identified in 15.2% of patients in the FT group and 22.7% in the SC group. All cases of secondary osteoarthritis were due to femoroacetabular impingement (Table 4).

**Table 4:** Patient characteristics.

Patient characteristics	FT	SC	*p* value
Number	46	44	
Sex	20 females and 26 males	15 females and 29 males	0.361
Age (years)	56.2 ± 8.5	54.2 ± 10.5	0.170
BMI	25.8 ± 3.4	30.2 ± 1.3	< 0.001
Smoke	26%	35%	0.328
Primary hip osteoarthritis	84.8%	77.3%	
Secondary osteoarthritis	15.2%	22.7%	

Abbreviations: BMI, body mass index; FT, fast track; SC, standard care.

### Outcome measures

Statistically significant differences were observed between groups in functional recovery and LOS. On postoperative Day 3, patients in the FT group showed greater independence, with a significantly lower mean ILOA score (9.60 ± 5.2) compared to the SC group (11.7 ± 3.4; *p *= 0.024, Mann–Whitney test), indicating faster early recovery of autonomy (Table 4).

Hospital length of stay (LOS) was significantly reduced in the FT group (3.54 ± 1.25 days) compared to the SC group (6.39 ± 1.59 days; *p *< 0.0001, Mann–Whitney test), reflecting the efficacy of the FT protocol in facilitating early discharge without compromising patient safety (Table 5). All patients in the fast‐track group were discharged home. In the SC group, two patients were transferred to rehabilitation centres, while the remaining patients were all discharged home.

**Table 5:** Length of hospital stay (days) and 3rd postoperative day ILOA score in FT and SC groups.

Parameter	Group	N. pts	Mean	SD	Lower limit CI	Upper limit CI	*p* value
ILOA score (Day 3)	FT	46	9.6	5.2	8	12.4	0.024
	SC	44	11.7	3.4	11.6	14.4	
Days of hospital stay	FT	46	4.0	3.1	3.2	3.8	<0.0001
	SC	44	6.4	1.6	6	7	

Abbreviations: CI, confidence intervals; FT, fast track; ILOA, Iowa level of assistance; SC, standard care; SD, standard deviation.

Functional outcomes, as measured by the WOMAC, were comparable between groups preoperatively (42.8 ± 10.6 for FT vs. 43.8 ± 8.9 for SC; *p* = 0.263). However, at 6 weeks postoperatively, the FT group reported significantly lower (i.e., better) WOMAC scores (10.38 ± 9.18) compared to the SC group (14.21 ± 8.76; *p* = 0.035). This trend persisted over time, with the FT group maintaining superior outcomes.

At 60 months, WOMAC scores were 0 ± 0 for the FT group versus 0.27 ± 0.81 in the SC group (*p* = 0.027) (Table 6). Preoperative HHS values were significantly higher in the FT group (70.79 ± 11.0) than in the SC group (61.0 ± 11.9; *p* = 0.001, ANOVA). The FT group consistently demonstrated superior postoperative HHS scores throughout follow‐up.

**Table 6:** WOMAC values in FT and SC groups during the follow‐up.

Scale	Group	N. pts	Mean	SD	*p* value
WOMAC preop	FT	46	42.8	10.6	0.263
	SC	44	43.8	8.9	
WOMAC 6 weeks	FT	46	10.38	9.2	0.035
	ST	44	14.2	8.8	
WOMAC 3 months	FT	46	2.8	7.3	0.145
	SC	44	5.3	8.4	
WOMAC 6 months	FT	46	1.2	4.5	0.052
	SC	43	2.2	4.8	
WOMAC 12 months	FT	45	0.3	0.2	0.057
	SC	43	0.5	1.2	
WOMAC 60 months	FT	42	0	0	0.027
	SC	41	0.27	0.8	

Abbreviations: FT, fast track; SC, standard care, SD, standard deviation WOMAC, Western Ontario and McMaster Universities Osteoarthritis Index.

Due to a statistically significant difference in baseline HHS values between the FT and SC groups, the Mann–Whitney *U*‐test was used to compare the change scores (i.e., improvement from baseline) in HHS between the two groups.

Results showed that the FT group demonstrated a lower mean improvement compared to the SC group at all time points. These differences reached statistical significance at:

6 months (*p* = 0.0037),

12 months (*p* = 0.0021),

and 60 months (*p* = 0.0017).

**Figure 2:**
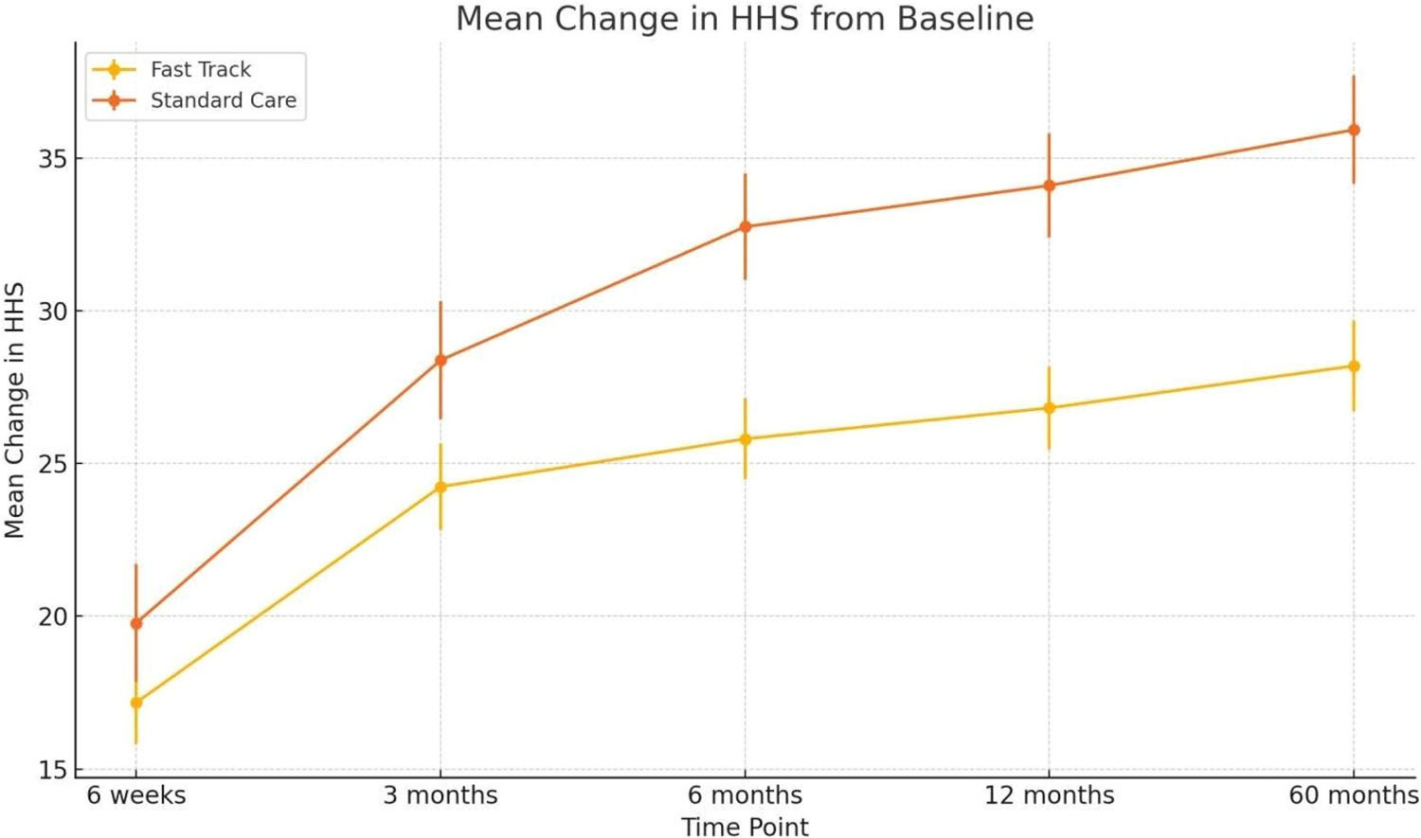
Mean change in HHS from baseline in FT and SC groups. FT, fast track; HHS, Harris hip score; SC, standard care.

Despite having higher baseline HHS values, patients in the FT group exhibited less improvement over time, likely due to a ceiling effect since their initial scores were already relatively high. These findings underscore the importance of adjusting for baseline differences when comparing the effects of interventions (Figure 2).

Hemoglobin levels were monitored preoperatively and during the first three postoperative days. No significant differences were observed between the groups at any time point.

### Complications

The most frequent postoperative complication in both groups was lateral femoral cutaneous nerve dysfunction, characterised by paresthesia. This occurred in four patients in the FT group and five patients in the SC group.

The FT group experienced one case of acute anaemia requiring transfusion, one intraoperative femoral fracture, and one instance of acetabular cup loosening. In contrast, the SC group reported one case of prosthetic hip dislocation and one case of psoas tendinopathy.

Additional adverse events included one periprosthetic joint infection in the FT group and one case of femoral nerve dysfunction in the SC group. Allogeneic blood transfusion was required in only one patient in the FT group, compared to five patients in the SC group, indicating a lower need for transfusion in the FT cohort (Table 7).

**Table 7:** Number and type of complications in the fast track and standard care groups.

Complication	Fast track group	Standard care group	*p* value
Acute anemia	1/46	0/44	0.325
Intra‐operative fracture	1/46	0/44	0.325
Cup loosening	1/46	0/44	0.325
Periprosthetic infection	1/46	0/44	0.325
Hip replacement dislocation	0/46	1/44	0.304
Psoas tendinopathy	0/46	1/44	0.304
Femoral nerve dysfunction	0/46	1/44	0.304
Total	4/46	3/44	0.676

## DISCUSSION

Our findings support the growing body of evidence demonstrating that FT protocols in THA significantly reduce hospital LOS and enhance early postoperative recovery without increasing complication or readmission rates. In our study, patients in the FT group achieved greater independence by postoperative Day 3 and were discharged earlier compared to those in the SC group, consistent with previous reports [12, 13, 14, 15, 16, 17, 18, 19, 25, 37].

The reduction in LOS observed in our FT group aligns with the outcomes of Husted et al. [14] and Morrell et al. [22], who reported that enhanced recovery pathways significantly shorten hospitalisation without negatively impacting patient safety. Moreover, Sutton et al. [33] showed that early discharge (within 0–2 days) was not associated with increased risks of major complications or readmissions, reinforcing the feasibility of shortened stays in appropriately selected patients.

Functional outcomes in our FT group, measured by the WOMAC score, were superior at both short‐ and long‐term follow‐up, with significant differences persisting up to 60 months but probably not clinical relevance. This is consistent with findings by Greimel et al. [12], who demonstrated improved early functional mobility in FT patients using objective performance measures such as the Timed Up and Go test.

However, although FT patients in our study had better absolute HHS at follow‐up, the magnitude of improvement was less than in the SC group, likely due to higher baseline scores and a potential ceiling effect. Similar results were reported by Maempel et al. [19], who found no significant difference in HHS improvement between enhanced recovery program and traditional care when adjusting for baseline differences.

These findings should also be interpreted in light of established thresholds for clinical relevance. In a study on THA, Singh et al. estimated the minimal clinically important difference (MCID) for the HHS to be approximately 15.9–18 points between the preoperative period and 2–5 years postsurgery [32]. Regarding the WOMAC, Quintana et al. reported mean improvements of ~37–39 points in the pain and stiffness domains at 6 months, with the MCID estimated at approximately 25.9 points for stiffness and 29.3 points for pain [28].

Therefore, although improvements in WOMAC and HHS were statistically significant in the FT group, they do not appear to reach the thresholds for clinical significance. It is also noteworthy that the FT group in our study started with higher preoperative HHS scores, which may have contributed to the smaller magnitude of change observed at the 5‐year follow‐up.

Importantly, the safety of FT protocols was reaffirmed in our study, with no increase in adverse events or transfusion requirements. This mirrors the findings of multiple studies [13, 14, 15] that have shown FT to be safe even in older or comorbid populations when preoperative optimisation and risk stratification are appropriately applied.

Our results also highlight the relevance of patient selection in FT implementation. In line with the work by Jørgensen and Kehlet [15], we limited inclusion to ASA I–II patients, minimising confounding from severe comorbidities. Nonetheless, literature suggests that even elderly and comorbid patients can safely benefit from FT protocols, provided adequate perioperative planning is in place.

Finally, the concerns about increased readmissions due to early discharge appear unfounded. As reported by Husted et al. [14], reduced LOS in fast‐track THA was not associated with a higher incidence of dislocations or thromboembolic complications. Similarly, our study observed no increase in adverse events, in agreement with the systematic review by Morrell et al. [22], which found no significant difference in morbidity or readmission between enhanced recovery and conventional pathways. The multidisciplinary approach is the driving force behind the FT protocol. It is not just a sequence of technical interventions, but a coordinated effort among various healthcare professionals (orthopaedic surgeon, anesthesiologist, nurse and physiotherapist), each with a specific and complementary role in patient management. The FT protocol demonstrates that the quality of care depends not only on the expertise of individuals but also on the synergy of the multidisciplinary team.

### Limitations

This study has several limitations. First, the single‐centre design may limit the generalisability of the findings to other clinical settings. Second, the exclusion of certain high‐risk populations—such as patients with high ASA scores, diabetes, BMI > 32, or rheumatoid arthritis—may reduce the applicability of the results to more heterogeneous patient populations. Comorbidities were not specifically analysed, although they are known to influence outcomes following THA [2, 20, 29, 31]. This limitation was partially addressed by including only patients classified as ASA I or II. Similarly, patients′ subjective perception of their hospital stay was not assessed, despite evidence suggesting its impact on functional outcomes and satisfaction [38]. We attempted to mitigate both of these limitations through the randomised allocation of participants to the study groups.

Another limitation is the inclusion of patients with secondary hip osteoarthritis, as well as significant between‐group differences in BMI and baseline HHS values between the FT and SC groups.

## CONCLUSIONS

This prospective randomised controlled study demonstrates that the FT protocol for THA had significantly lower mean ILOA scores on postoperative Day 3 and a shorter LOS compared to SC. Patients in the FT group achieved earlier independence, as evidenced by lower ILOA scores at postoperative Day 3 and were discharged home more rapidly compared to those receiving SC.

Functional outcomes, measured by WOMAC and HHS, favoured the FT group throughout the follow‐up period, with significantly better WOMAC scores at 6 weeks and sustained superior performance up to 5 years postoperatively. Although the clinical relevance of this data is questionable given the negligible absolute difference. Furthermore, there is an imbalance in terms of BMI and HHS in the two groups that could affect final long‐term results, which must be interpreted carefully. After adjusting for baseline differences, the magnitude of improvement in HHS was lower in the FT group, likely reflecting a ceiling effect due to their higher preoperative functional status.

These findings underscore the efficacy of the FT approach in optimising postoperative recovery in selected patients undergoing THA. Future multicenter studies with larger and more heterogeneous populations are warranted to confirm the generalisability of these results and to further refine patient selection criteria.

## AUTHOR CONTRIBUTIONS

**Pietro Cimatti**: Study conceptualisation, methodology, investigation, writing original draft, manuscript review and editing, **Martina Rocchi**: Patient enrolment, methodology, investigation, writing original draft, manuscript review and editing, **Benedetta Dallari**: Software, formal analysis, validation, manuscript review and editing, **Nicolandrea Del Piccolo**: Resources, visualisation and supervision, manuscript review and editing, **Alessandro Mazzotta**: Investigation, data curation, resources, manuscript review and editing, **Dante Dallari**: Supervision, project administration, manuscript review and editing. All authors read and approved the final version of the manuscript.

## ETHICS STATEMENT

The study was approved by the Institutional Review Board (IRB) of Istituto Ortopedico Rizzoli. The study protocol was registered in ClinicalTrials.gov with the number NCT03875976. Written informed consent was obtained from all participants before their inclusion in the study. Written informed consent was obtained from all patients for participation in the study and for publication of any associated data and images.

## Data Availability

Data available on request from the authors.
